# A study protocol for neonatal sepsis and gut microbiomics among preterm infants admitted at Muhimbili National Hospital, Tanzania

**DOI:** 10.1371/journal.pone.0325099

**Published:** 2025-06-02

**Authors:** Fatima M. Mussa, Agricola Joachim, Robert Moshiro, Salim S. Masoud, Marylyn M. Addo, Julia Pagel, Ralf Krumkamp, Robin Kobbe, Nahya Salim

**Affiliations:** 1 Department of Pediatrics and Child Health, School of Medicine, Muhimbili University of Health and Allied Sciences, Dar es Salaam, Tanzania; 2 Department of Microbiology and Immunology, School of Diagnostic Medicine, Muhimbili University of Health and Allied Sciences, Dar es Salaam, Tanzania; 3 Department of Pediatrics and Child Health, Muhimbili National Hospital, Dar es Salaam, Tanzania; 4 German Centre for Infection Research (DZIF), partner site Hamburg-Lübeck-Borstel-Riems, Germany; 5 Institute for Infection Research and Vaccine Development (IIRVD), University Medical Center Hamburg-Eppendorf, Hamburg, Germany; 6 Department of Pediatrics, University Hospital Hamburg-Eppendorf, Hamburg-Eppendorf, Germany; 7 Department of Infectious Disease Epidemiology, Bernhard Nocht Institute for Tropical Medicine, Hamburg, Germany.; Hawassa University College of Medicine and Health Sciences, ETHIOPIA

## Abstract

**Background:**

Neonatal mortality remains high in many low- and middle-income countries (LMICs), with neonatal sepsis and antimicrobial resistance (AMR) posing significant threats to newborns, particularly in sub-Saharan Africa (SSA). Tanzania is among the countries with the highest neonatal mortality rates, with sepsis being a major contributor.

Gut dysbiosis has been identified as a risk factor for neonatal sepsis in high-income countries, due to factors like abundance of pathogenic bacteria, decrease in microbiome diversity, intestinal barrier defects and bacterial translocation. Understanding gut dysbiosis in the local setting and its role in sepsis development may offer new prevention strategies, such as probiotics for high-risk preterm infants.

**Objectives:**

This prospective neonatal cohort, established at Muhimbili National Hospital (MNH) in Dar es Salaam, Tanzania, aims to analyze the gut microbiome of preterm infants and explore associations with neonatal late-onset sepsis (LOS). Additionally, data on bacterial pathogens of bloodstream infections and AMR prevalence will be identified. Secondary endpoints include clinical LOS, sepsis-related death, death from any cause, and hospital discharge outcomes.

**Methods:**

Eligible preterm neonates (28 + 0 to <34 weeks of gestational age, birth weight ≥ 1000g) will be recruited with maternal consent. Socio-demographic and clinical data, microbiological details of blood pathogens, and a set of fresh frozen fecal samples during the 28 days observation period will be collected. The study targets a sample size of 1350 participants and we expect 72–135 culture-proven LOS during a study period of 18 months. Fecal samples will undergo next-generation sequencing (NGS) to analyze microbial community functions in comparison to matched controls.

**Discussion:**

This collaborative study between universities in Tanzania and Germany, aims to analyze the neonatal microbiome in relation to sepsis development and AMR of blood culture isolates to enhance neonatal sepsis care, improve diagnostics and treatment. The project will offer insights into potential therapeutic strategies for the future, promote academic exchange, capacity building and research on African microbiomes.

## Introduction

Despite significant progress in reducing child mortality, neonatal mortality remains alarmingly high in many low-and middle-income countries (LMICs). One of the major global contributors to neonatal mortality is sepsis [1], which is further complicated by the increasing antimicrobial resistance (AMR) of pathogens [2,3]. It is vital to understand the epidemiology of neonatal sepsis and AMR at local, regional, and national levels in LMICs to develop effective treatment and prevention strategies.

Tanzania is among the countries with the highest newborn mortality in sub-Saharan Africa (SSA), with sepsis being one of the major causes of death [4]. The Tanzanian government, through the Ministry of Health, launched a strategic plan to accelerate the reduction of maternal and newborn deaths and is currently working to establish well-equipped and properly staffed neonatal units in all regional referral and district hospitals [5,6]. The need assessment for this project was based on opinions from international experts, as well as results from local studies done on neonatal sepsis [7–10].

Recent advances in neonatal care services, the establishment and improvements in neonatal care units (NCU) in Tanzania have seen even more preterm and very low birth weight (VLBW) neonates surviving the first weeks of life. However, VLBW neonates also have the highest risk for late-onset sepsis (LOS) and necrotizing enterocolitis (NEC) with high morbidity and mortality and serious short and long-term health consequences [8]. A recent multicenter global study conducted in LMICs reported that 21% of VLBW neonates experience at least one episode of blood culture-proven LOS [10]. Various factors are associated with intestinal inflammation and bacterial translocation into the bloodstream, including their intestinal barrier immaturity, their premature immune system, and gut microbiota dysbiosis [11].

In resource-limited clinical settings, identification of pathogens causing neonatal sepsis and analysing AMR profiles remain challenging. Isolated bacteria are increasingly resistant to first and second-line antibiotics recommended by the WHO [12,13]. Extended-spectrum beta-lactamases (ESBL)-positive *Klebsiella* and *E. coli* spp. are reported to become the most common causes of neonatal sepsis in SSA with rapid emergence of carbapenem-resistant *Enterobacterales* [14]. New and affordable antibiotics for the treatment of multi-drug resistant (MDR) neonatal sepsis are lacking [15], therefore, in addition to the development of new drugs, infection prevention control (IPC) is pivotal.

Establishing reliable sepsis and AMR diagnostics through blood cultures of neonates admitted to NCUs is crucial for identifying bloodstream infections and determining antimicrobial susceptibility, allowing clinicians to prescribe antibiotics more efficiently and appropriately. A large multi-country study, including our study site, published by NEST360 in 2023, highlighted a significant gap: while 70% of neonates were prescribed antibiotics, only 6% had blood cultures taken, missing critical opportunities for infection detection [16]. The authors concluded that there is an urgent need to improve diagnostics, and develop individualized strategies for the prevention and treatment of LOS in LMICs.

The peak incidence of LOS occurs between the second and third week of life [17] after being exposed to various factors in the NCU environment and exposures to various invasive procedures and antibiotics altering the neonatal microbiome [18]. The hypothesis of gut dysbiosis preceding the development of LOS and NEC in preterm babies has been developed, based on findings of alterations in their gut microbiome in terms of specific metabolites and reduced biodiversity before disease onset [19–21]. Importantly, several conventional culture studies have demonstrated concordance between isolates cultured in blood and bacteria residing in the infants’ gut [22,23].

Recent advances in molecular microbiology and bioinformatics including next-generation sequencing have enabled direct and deep sequencing of bacterial DNA from stool samples of neonates to describe the gut microbiome, identify dysbiosis, and investigate its impact on the risk of developing sepsis and AMR transmission. The aim of this study is to analyze maternal and neonatal gut microbiome and the risk to develop LOS or NEC. This collaborative academic partnership study is, to our best knowledge, the first to examine the gut microbiome of preterms in Tanzania.

### Research questions

Our primary research question aims to answer whether gut dysbiosis precedes the development of LOS and/or NEC in Tanzanian preterm neonates and whether microbial or metabolomic markers predict neonates at high risk. Secondary research questions include 1) What are the maternal and neonatal risk factors for LOS and/or NEC? 2) What are factors associated with disease severity and outcomes? 3) What bacteria species and AMR profiles are found in blood culture isolates of preterm neonates with LOS and/or NEC?

## Materials and methods

### Study design

This study will be a case-control study nested into a hospital-based, single-center, observational neonatal cohort. Recruited preterm neonates will be followed over their first 28 days of life. The primary clinical endpoint in this study is a blood culture (BC)-proven LOS. Secondary endpoints are clinical sepsis, NEC, death to clinical and/or BC-proven sepsis or NEC, death to any cause, or discharge from the hospital with or without sequelae.

### Study setting

The study will be conducted at the Muhimbili National Hospital (MNH) in Dar es Salaam, the business and commercial center of Tanzania. MNH is the country’s largest and highest referral care center receiving newborns throughout the country, specifically from all regional referral sites. MNH is the teaching hospital for the Muhimbili University of Health and Allied Sciences (MUHAS) and other medical universities.

The Neonatal Care Unit (NCU), located near the obstetric theater and maternal wards, includes the preterm unit (Ward 37), term unit (Ward 36), and Kangaroo Mother Care (KMC) unit, with a total capacity of 137 beds, including 14 NICU beds equipped with 10 mechanical ventilators. The High Dependency Unit (HDU) accommodates 10 neonates in warmer beds with CPAP support. Over the past four years, the unit has been upgraded to a level III NICU – one of only four in the public sector in the entire country – through significant investments in infrastructure, equipment, and staff training. The introduction of the Master of Science in Clinical Neonatology program in 2021 has further enhanced preterm care standards by training pediatricians as specialized neonatologists.

The NICU and HDU beds usually serve as stations for the sick preterm admissions before transitioning to Ward 37 for completion of treatment or further observation, after which they are either discharged home or referred to the close-by KMC unit for weight monitoring and feeding progression. The discharge criteria for preterm neonates include reaching a minimum weight of 1.5 kg, consistent weight gain of 15–30 g/kg/day, being in stable health, and receiving full enteral feeds either by cup or breastfeeding. After discharge, preterm infants are routinely followed up weekly at the outpatient neonatal clinic until they reach a weight of 2.5 kg. The NCU receives approximately 4000 neonatal admissions per year. Specifically, around half of these (150–180 admissions per month) include prematurely born babies with gestational age (GA) of less than 34 weeks.

### Study population and sample size

The study participants will include inborn and outborn preterm neonates born at 28 + 0 to <34 weeks of GA admitted to the neonatal wards, and their respective mothers who consented to participate after a detailed description of the study has been given by trained study team members. Recruited preterm neonates should have a minimal weight of at least 1000 g and be enrolled within their first week of life. We will exclude infants whose mothers are below 18 years of age, have severe, life-threatening illness at the time of admission, and/or have severe comorbidities or severe congenital malformations. Furthermore, neonates or mothers who ever received probiotic supplements during pregnancy will be excluded from this study.

Sample size calculations considered all study objectives and the one requiring the largest sample size will be the basis to determine recruitment numbers. All eligible preterm neonates admitted to the neonatal unit during the 18-month study period will be recruited and closely followed up for 28 days each.

To answer the primary research question on the hypothesis of preterm neonates developing gut dysbiosis before the onset of sepsis we need to identify preterms with BC-proven sepsis, assess their microbiomes, and compare it with controls (matched for GA, sex, mode of delivery, type of feeding, and previous antibiotic exposure). Assuming a rate of BC-proven sepsis of 10% and 25% missed samples we expect to identify 72–135 bloodstream pathogens (positive BCs) with 54–101 complete sets of stool samples for microbiome analysis within 18 months [7,10]. According to Graspeuntner *et al.,* we then expect gut dysbiosis in 30% of the neonates without and 60% of the children with LOS [19].

Our minimal sample size considerations are based on the proportions of gut dysbiosis in children with and without LOS. Assuming an alpha level of 5% and a power of 80% a minimal study number of 84 children (42 with and 42 without LOS) is needed to reject the null hypothesis that no difference in the proportion of dysbiosis between both cases and controls. Cases are defined as preterm neonates who develop LOS and controls as preterm neonates who do not develop any clinical or culture proven sepsis during the observation period (after 72 hours of life).

The calculations are based on formulae 1 for a two-sample proportions test, where *n*_*i*_ is the sample size estimated for each group, z is the normal deviation defined for the alpha level (α) and the study power (β), and *p*_*i*_ is the proportions in the respective groups.


$$n_{i}=\frac{{\left(Z\alpha /2+Z1-\beta \right)}^{2}*(p1(1-p1))+(p2(1-p2))}{{\left(p1-p2\right)}^{2}}$$ (formulae 1)

These parameters define minimal study numbers to reach power and significance. We aim to include all cases of LOS and NEC with complete microbiome and a higher number of controls, to improve the statistical power of the study, precision of the applied data analysis and allow potential subgroup analyses [19].

In the literature, the rate of positive BCs varies between study sites and is highly dependent on the quality of diagnostic procedures as well as the amount of blood transferred into BC bottles. Based on the available data and our experiences we are expecting to screen 80–100 admissions per month in the respective GA range and a study inclusion rate of 50–75%. Thus, we expect to recruit approximately 40–75 admitted newborns per month for a period of 18 months, which amounts to 720–1350 admissions over the study period. The calculations were enforced by comparing data from previous studies [7,24] and the ongoing NEST360 survey in the NCU [25], powered to identify sepsis incidence, risk factors, and mortality rates of LOS and NEC among preterm neonates.

### Definitions of LOS, culture proven sepsis, clinical sepsis and NEC

LOS is defined as a bloodstream infection occurs after 72 hours of life, typically caused by nosocomial or community-acquired pathogens [26]. Culture-proven sepsis is identified when a bacterial or fungal pathogen is isolated from a sterile site, such as blood or cerebrospinal fluid. In contrast, clinical sepsis is diagnosed based on clinical signs and symptoms consistent with sepsis in the absence of a BC [27]. The signs and symptoms of neonatal sepsis range from nonspecific manifestations to hemodynamic collapse, requiring a high index of suspicion for timely diagnosis. This study will uniformly apply the WHO criteria for neonatal sepsis, which offer a standardized approach for identifying sepsis in preterm neonates [28]. LOS will be defined by the following criteria by responsible physicians, at least the presence of two signs as per WHO which includes difficulty feeding, convulsions, lethargy, tachypnoea, severe chest indrawing, hypothermia, hyperthermia, bulging anterior fontanelle and skin or umbilical infection with pus requiring systemic antibiotics for at least five days [28].

Cases will be categorized as culture-proven sepsis and clinical sepsis. Instances of positive BCs without accompanying signs or symptoms will be considered contaminants. Specifically, coagulase-negative staphylococci (CoNS) will be classified as a pathogen only if overt signs and symptoms consistent with sepsis, as defined by WHO criteria, are present; otherwise, CoNS will be regarded as contaminants [29]. To improve diagnostic accuracy where available, C-reactive protein (CRP) levels will be used as a biomarker during suspected sepsis episodes, with elevated levels supporting the presence of an inflammatory response [30].

In this study, NEC is defined and suspected clinically based on the presence of one or more of the most characteristic clinical features including abdominal distension, feeding intolerance, bilious vomiting or gastric aspirate and bloody stools, and if available, complemented by the finding on abdominal imaging of intramural gas, pneumoperitoneum or sentinel bowel loops [31,32].

### Variables to predict LOS and gut dysbiosis

Variables have been selected based on a literature search and local clinical experience. Table 1 shows factors predicting LOS and gut dysbiosis that will be captured during the study period. Independent variables will be categorized as socio-economic, neonatal, maternal, and septic risk factors (Table 1).

**Table 1:** Candidate variables for predicting LOS and gut dysbiosis.

Socio-economic factors	Neonatal factors	Maternal factors	Septic risk factors
Maternal education level	Gestational age	Age at delivery	Invasive ventilation
Self-rated household income	Sex	Parity	Central line at least 48 hrs
Family health insurance	Birth weight	Previous stillbirths	Parenteral nutrition
	Multiple births	HIV status	Presence of any maternal risk factors
	APGAR score at 1 minute	Medical conditions	Type of feeding
	APGAR score at 5 minutes	Pregnancy complications	
	Place of birth	Maternal antibiotic exposure 3 months before delivery	
	Delivery complications		
	Antibiotic exposure and type		
	Type of delivery		

During the observational period of 28 days clinical signs and symptoms of LOS and/or NEC, BC results with phenotypic identification, and antibiotic susceptibility testing, the previous use and type of antibiotics, and the clinical outcomes, will be recorded by the study team in electronic data capture forms. Stool samples for microbiome analysis will be collected in a standardized manner on day 1–3, and days 7, 14, 21 (each ±3) and neonates’ type of feeding in the last 24 hours will be categorized as exclusive human milk (no formula given), breastfed mainly (>80% feeds breastmilk/human milk), formula feed (>50% of feeds include formula), only formula (no human milk), or no enteral feeding at all.

### Data collection team, study period and timelines

Data will be collected by a dedicated study team, consisting of a local medical officer and two study nurses (trained nurse attendants), under the coordination and supervision of a study coordinator – a pediatrician working in the neonatal unit. To ensure seamless collaboration, all neonatal unit staff will be informed and educated about the clinically based survey. This collaborative approach will support effective recruitment, sampling, and adherence to national treatment guidelines, ultimately enhancing clinical care. The study began on October 23, 2023, and is planned to run for two years. Participant recruitment will span 18 months, followed by six months dedicated to stool sample processing and gut microbiome DNA sequencing. Analysis of clinical data and microbiome profiles will commence immediately afterwards.

### Data collection tools and methods

All study data will be pseudonymously recorded and collected using password-secured tablets and managed using REDCap^®^ electronic data capture tools [33,34]. Patients will be screened for inclusion and if recruited a copy of the study admission form will remain in the patient`s file to avoid re-recruitment. Study team members will enter the data at recruitment, at times of sample collection, during each episode of sepsis or NEC, and discharge/referral or death, including information from microbial reports and other laboratory results into the eCRFs. At the end of follow-up all co-morbidities and complications will be evaluated and recorded. All data points will be checked by another study member, validated and approved by the study coordinator or local PI.

### Demographic and clinical parameters

Properly designed eCRFs will be used to collect relevant information from the mother and the preterm neonate. Information will be obtained from the maternal medical records, admission form, direct interview with the mother, and examination of the preterm neonate on admission and during their hospital stay. Maternal medical charts will be reviewed for the GA estimation using the best available information either the last menstrual period (LMP) or early obstetric ultrasound, whichever is available. The participants’ weight at admission and throughout the observation period will be measured using a calibrated SECA^®^ medical scale (UK), with readings recorded to the nearest whole number in grams. The scale will be calibrated daily to ensure accuracy. Concurrently we will perform a New Ballard Score (NBS) for those preterms recruited within 48 hours to confirm the GA recorded. In addition, we will take the foot length during NBS examination to analyse it as a proxy for GA assessment in comparison to valid data like the early obstetric ultrasound or LMP.

Once recruited, initial demographic, pregnancy, and birth history, maternal risk factors will be recorded and the preterm neonate be followed up daily for 28 days after birth for routine newborn care and any of the outcomes of this study. If the preterm neonate is discharged before the 28-day follow-up period, all attempts will be made to monitor them during their scheduled weekly outpatient clinic visits, where they will undergo clinical assessments for any signs of sepsis. If sepsis is suspected, they will be readmitted for appropriate care. At day 28 of life, a phone call will be made to those that were prematurely discharged to assess if the infant is alive and if there was any readmission due to suspected sepsis.

### Samples collection

#### Blood cultures.

To describe the etiological agents and AMR patterns of neonatal bloodstream infections, we will be collecting 1–2 ml sterile blood for cultures [35] in BD BACTEC™ Peds Plus BC bottles according to international recommendation guidelines in all participants developing signs and symptoms of LOS or NEC [36], without delaying antimicrobial treatment. For participants at risk of developing early onset sepsis (EOS), termed as presumed EOS according to standard treatment guidelines, and started on antibiotics based on risk factors, BCs will also be taken free of charge before initiation of treatment whenever possible. Another BC will be taken in cases of switching, adding, or escalating antibiotic therapy. An episode of sepsis will be classified as culture-proven sepsis or clinical sepsis depending on BC results and additional clinical information by the study physicians. The blood culture samples will be processed in the microbiology laboratory at MNH which is certified in accordance with the international standard (ISO 15189:2022).

#### Isolation and identification.

BC bottles that have been inoculated will be sent to the laboratory as soon as possible by the study nurses, or stored at ambient temperatures if transport to the lab is delayed. Once BC bottles are received in the microbiology laboratory, registration follows whereby BC bottles are given the unique ID number and loaded into the automated BACTEC^®^ system (BACTEC® 9050; Becton Dickinson) for incubation and monitored for a maximum of five days to observe bacteria growth. For each positive BC, a primary gram stain will be performed and communicated to the clinician as soon as possible. Organisms will be sub-cultured on Blood Agar (BA), MacConkey (MCA, Oxoid, UK), and or Sabouraud Dextrose Agar (SDA) if the primary gram stain observed the presence of budding yeast cells microscopically. Organisms will be identified based on their colonial morphology and biochemical characteristics as described by Murray et al. (30). Gram-negative organisms will be identified using oxidase, Kliger Iron Agar (KIA), sulfur indole and mobility (SIM), a urease, and citrate test, while gram-positive organisms will be detected by catalase reaction, coagulase test, DNase test, and bile esculin test (30). Analytical Profile Index (API) 20E and API-20 NE (BioMérieux, France) tests will be used to confirm isolates in case of ambiguity.

#### Antimicrobial susceptibility pattern.

Antimicrobial susceptibility testing (AST) of the bacterial isolates will be done by the conventional Kirby-Bauer disk diffusion method as per Clinical Laboratory Standard Institute (CLSI) guidelines. AST will be done for antibiotics that stand as the first, second, and third line agents used to treat neonatal sepsis in the Tanzania treatment guidelines and used at MNH; which are ampicillin, cloxacillin, aminoglycosides (gentamycin and amikacin), and cephalosporins (cefotaxime, ceftriaxone and/or ceftazidime), fluoroquinolone (ciprofloxacin), amoxicillin-clavulanic acid, piperacillin-tazobactam, and carbapenem (meropenem). Ceftriaxone/Sulbactam will be tested using an E-test for Gram-negative bacteria. In the case of Gram-positive bacteria, it is recommended to use vancomycin if all tested antimicrobial agents are resistant. In addition, the combination disc diffusion method will be performed for detection of ESBL-producing Gram-negative organisms, Inducible Clindamycin Resistant (ICR) and MRSA/MRCoNs also tested for Gram-positive isolate according to CLSI guidelines. AST results will be reported as sensitive, intermediate, and resistant. Intermediate results will be considered resistant during analysis. Blood culture and AST results will be recorded, including clinical information as described in the eCRF. These results will be immediately communicated to the attending physician for management of the preterm infants.

#### C-reactive Protein.

During a sepsis episode, C-reactive protein (CRP) will be taken as part of the laboratory workup using the Architect c8200 clinical chemistry analyzer. CRP result >10mg/l will be considered high and indicative of infection/inflammation.

When suspecting early-onset sepsis, CRP is routinely taken 48 hours after birth as part of the SOP of the NCU, as one of the parameters used to guide antibiotic treatment. If the CRP is high, antibiotics will be continued or changed depending on the clinical condition of the neonate while awaiting the BC results. If it is low, and the patient is clinically well and BC is negative, antibiotics are stopped and sepsis is ruled out.

For LOS, CRP is usually taken during or close to the sepsis episode and is interpreted as one of the diagnostic markers. The CRP taken during or closest (within 48 hours) to the sepsis episode will be recorded.

#### Gut microbiome analysis.

To investigate gut fresh fecal samples will be obtained from soiled diapers in disposable sterile tubes, as fresh as possible, and preserved using PowerProtect^®^ DNA/RNA buffer (QIAGEN GmbH, Hilden, Germany) as per the manufacturer’s guidelines. Following SOPs within 20 minutes of collection samples will be placed in a cooling box maintained at temperature of 4–6°C, and transported to the Central Pathology Laboratory at MNH, which is about 7 minutes’ walk where they will be stored in a −80°C freezer within 4–6 hours. The mother will also be asked to provide a stool sample within the first few days postpartum using the same collection and storage protocols. DNA extraction from samples will be performed at MUHAS microbiology laboratory and an aliquot of frozen DNA samples will be transferred to Germany for shotgun metagenomics (for details please see S1 File), all in compliance with national regulations (NIMR Material Transfer Agreement) and international guidelines (Nagoya Protocol). Necessary permissions for sample transfer will be strictly adhered to. Samples will be stored for 5 years after completion of the study before destruction. Data will be kept for 30 years according to international standards.

### Investigation tools’ validity and reliability of the data

All data collection tools will undergo pre-testing during a pilot phase involving at least ten participants before the full study begins. Before the project launch, the entire unit will be briefed on the study’s aims and objectives to ensure that all staff adhere to uniform procedures and standards of care for the vulnerable populations involved. Research assistants will receive training in Good Clinical Practice (GCP), research ethics, and the proper use of study tools, including the REDCap^®^ data management system, to ensure accurate data collection. Continuous training on hygiene, aseptic techniques for drawing and storing blood culture samples, and infection prevention measures will be conducted to minimize contamination and maximize sample yield. Data quality will be regularly monitored by the PIs, study managers, and data managers through reports, data exports, and statistical checks to maintain consistency. All data entries will be cross-checked by another study team member and validated by the study coordinator. A robust data management system led by experienced data managers and the research team will be used to clean and export data for analysis in R statistical software, ensuring the accuracy and integrity of the collected data.

Data entry will be performed by authorized research staff using touch-screen tablets equipped with the REDCap^®^ data management system to allow real-time data entry. Additionally, a customized Android application for the tablets will be utilized locally to manage participant registration, appointment schedules, and sample storage. Entered data will be securely transmitted to servers located at MUHAS and the German site. These main servers will only be accessible to the data managers and PIs on request.

For data validation and resolution, the data management team will conduct monthly reviews using established quality checks to identify discrepancies, missing fields, or logic errors in the collected data. Any issues found will be communicated to the research team for resolution. An automated audit trail will track all corrections, ensuring transparency and accountability throughout the data management process.

### Statistical analysis

Descriptive statistics will be used to summarize the demographic and clinical characteristics of the study population, including gestational age, birth weight, gender, mode of delivery, and presence of comorbidities. Mean and standard deviation or median and interquartile range for continuous variables will be used and for categorical data frequencies or proportions will be described.

To answer the first objective on the incidence and risk factors of LOS, the incidence density will be calculated as the number of new cases of LOS divided by the total person-time at risk in the cohort. The person-time at risk will be calculated by summing the individual follow-up durations until the occurrence of BC-proven LOS (clinical sepsis, NEC, and death).

Cox-proportional hazard regression will be utilized to investigate the relationship between the risk factors and risk to develop primary and secondary endpoints. First, we will perform univariate cox-regressions for potential risk factors to identify individual risk factors, all variables considered relevant for the study question will be included in the multivariate cox-regression to adjusting for potential confounders. The hazard ratios (HRs) with the 95% confidence intervals (CIs) will be estimated for each risk factor. Etiological agents of LOS/NEC and the respective AMR patterns will be described as the proportion of isolates resistant to each antibiotic and according to international standard.

Microbiome analysis will be performed to evaluate gut dysbiosis in preterm infants prior to the development of LOS and in relation to the microbiome of the mother. Association of relative abundances of microbial species with the factors of gestational age, days of life, diet, and LOS will be limited to species that were found with a non-zero relative abundance in at least 40 stool metagenomes. Square root-transformed relative abundancies will be used and analysed using linear mixed effect (LME) models, where repeatedly collected microbiome data from each child is considered to cluster.

Antibiotic interventions following a LOS diagnosis are likely to have a strong impact on the microbiome composition. Statistical analyses will be performed using the latest version of R statistical software. Cox regression will be calculated using the *survival* package and LME using the *lme4* package. In all above-mentioned association tests, samples taken after LOS diagnoses will be excluded from the statistical analysis since the present study focusses on the microbial pattern that precedes LOS.

### Ethics approval, ethical issues and consent to participate

The study will be conducted in accordance with the ethical principles of the Declaration of Helsinki and has received local ethical approval from Muhimbili University of Health and Allied Sciences institutional review board with ethical clearance number 06-2023-1764, nationally by the National Institute for Medical Research (NIMR) with clearance certificate number NIMR/HQ/R.8a/Vol.IX/4407, and 2023–101040-BO-ff, by the responsible German approval of the Ethik-Kommission der Ärztekammer Hamburg, Germany. The study is registered at the Pan African Clinical Trial Registry (pactr.samrc.ac.za) with the unique identification number PACTR202502518189291.

Permission to collect data will be obtained from the research committee at Muhimbili National Hospital. An informed written consent to participate in the study will be sought (through signature) from the mother before recruitment. For those who are unable to read, the study team will explain to them all the components of the study in the presence of a witness and a thumb print will be taken prior to enrolment. No minors will be enrolled in this study due to adherence to German Pharmaceutical Act (AMG) §§ 40 IV and 41 II. All the preterm babies identified with a resistant organism will be communicated immediately and managed as per national guideline of Tanzania and MNH, standard procedures will be adhered to ensure receipt of appropriate antibiotics. Ownership and access to all samples and data is shared between the partners and regulated by signed sample and data transfer and sharing agreements.

### Inclusivity in global research

Additional information regarding the ethical, cultural, and scientific considerations specific to inclusivity in global research is included in the Supporting Information. (S2 Checklist).

## Discussion

Neonatal sepsis is one of the leading cause of neonatal mortality and long term morbidity especially among preterm infants in LMICs. Our collaborative study aims to generate informative data on LOS and AMR in Tanzania, improve the quality of diagnostics and treatment of sepsis and overall neonatal care. The systematic collection of data offers the opportunity for targeted antimicrobial therapy and improved clinical outcomes for neonates. As an academic hospital partnership study the activities will help scale up and conduct more, possibly multi-site, research projects in neonatal sepsis. This approach aligns with reducing neonatal mortality as a key target to achieving the Sustainable Development Goal number 3.2. Knowledge and experience exchange between academic partners and associated research groups will hopefully help to improve the outcome of neonates over the coming years. The study will also provide crucial local microbial monitoring data tailored to our specific setting to optimize diagnosis and treatment, paving the way for additional clinical research projects and integration into national and international surveillance networks. Furthermore, support for the microbiology laboratory, coupled with training and research exchanges will strengthen laboratory capacities and, will contribute to overall quality improvement at the hospital level.

Our translational research approach of microbiome sequencing and biostatistical analysis aims to identify additional markers and risk factors for LOS and NEC. It offers new opportunities for Tanzanian researchers. While research in high income countries supports the hypothesis that dysbiosis in the gut prior to development of LOS is an additional risk factor [19], little is known about the composition of the neonatal microbiome in the LMIC setting where the burden of sepsis in the highest. This study will hopefully generate a better understanding of the associations of microbiome characteristics and risk of sepsis in this population of preterm infants. Results may be useful for discussing possible benefits of therapeutic or prophylactic targeted treatment. Identification of metabolomic molecules could, for example, lead to a more comprehensive diagnostic approach of specific biomarkers, which could be established on a local metabolomics platform like High Performance Liquid Chromatography (HPLC) or photometer. Microbiome modification with probiotics is already used in high-resource settings as novel microbiota-based treatment strategies, that could be explored further in the African healthcare environment to improve outcomes of neonatal LOS and NEC. Thus, this cohort study complements the AMR surveillance work by looking at sepsis from another dimension of the gut microbiome, that could pave the way for additional research and preventive therapies in the future.

## Supporting information

S1 File Supplementary material on gut microbiome analysis. (DOCX)

S2 Checklist Inclusivity in global research questionnaire. (DOCX)

## Abbreviations

AMR: Antimicrobial resistance
AST: Antimicrobial susceptibility testing
BC: Blood culture
CLSI: Clinical Laboratory Standard Institute
CoNS: Coagulase- negative Staphylococci
CPAP: Continuous positive airway pressure
CRP: C-reactive protein
eCRF: Electronic Case Record Form
EOS: Early onset sepsis
ESBL: Extended spectrum Beta lactamase
GA: Gestational age
GCP: Good clinical practice
HDU: High dependency unit
HR: Hazard Ratio
HPLC: High Performance Liquid Chromatography
ICR: Inducible Clindamycin resistance
KMC: Kangaroo mother care
LMIC: Low and Middle income countries
LOS: Late onset sepsis
MDRO: Multi-Drug resistant organisms
MNH: Muhimbili National Hospital
MRCoNS: multi-resistant coagulase-negative staphylococci
MRSA: Methicillin resistant staphylococcus aureus
MUHAS: Muhimbili University of Health and Allied Sciences
NEC: Necrotizing enterocolitis
NGS: Next Generation Sequencing
NCU: Neonatal care unit
NICU: Neonatal intensive care unit
NIMR: National institute for medical research
PI: Principal investigator
SDGs: Sustainable development Goals
SOP: Standard operating procedures
SSA: Sub-Saharan Africa
